# Accuracy of Nutrition-Related Awareness Messages on Twitter (Rebranded as X) by the Nutrition Awareness Providers in the Kingdom of Saudi Arabia: Validity Content Analysis

**DOI:** 10.2196/68128

**Published:** 2025-09-26

**Authors:** Duaa Alammari, Iman A Bindayel, Noura Althukair, Noura AlRomi, Khalid Aldubayan, Najla Khateeb, Ahmed Alabdrabalnabi, Banan Banamah

**Affiliations:** 1 Department of Health Systems Management, College of Public Health and Health Informatics, King Saud Bin Abdulaziz University for Health Sciences, Prince Mutib Ibn Abdullah ibn Abdulaziz Rd, Riyadh, 13213, Saudi Arabia, 966 500844881; 2 King Abdullah International Medical Research Center, Riyadh, Saudi Arabia; 3 Department of Community Health and Sciences, College of Applied Medical Sciences, King Saud University, Riyadh, Saudi Arabia; 4 College of Applied Medical Sciences, King Saud Bin Abdulaziz University for Health Sciences, Al Ahsa, Saudi Arabia; 5 Department of Public Health, College of Health Sciences, Saudi Electronic University, Dammam, Saudi Arabia

**Keywords:** nutrition, awareness, content analysis, social media, twitter (X), nutritional, dietary, nutrition awareness, Asian, Saudi Arabian, media, health information, mixed methods, clinical nutrition, food science

## Abstract

**Background:**

With the increasing use of social media, platforms like Twitter (rebranded as X in 2023) have become popular channels for disseminating health information. In Saudi Arabia, Twitter is widely used, making it an effective tool for health awareness. However, the accuracy of nutrition-related content on social media is often questioned.

**Objective:**

The study aims to evaluate the accuracy and evidence-based quality of nutrition-related tweets posted by reputable Saudi health and nutrition awareness providers.

**Methods:**

A mixed methods content analysis was conducted on tweets published by 7 Saudi health organizations, examining content in Arabic and English over 12 months. Nutrition-related tweets were analyzed for accuracy, popularity, and evidence inclusion by a panel of experts in clinical nutrition, food science, and technology.

**Results:**

A total of 531 nutrition-related tweets were included in the study. Findings indicate that 445 (84%) of the tweets were accurate, of which only 17 (4%) included cited evidence. Yet, only 13 (2%) were inaccurate. The highest number of tweets are from Saudi Food and Drug Administration (SFDA) 96 (18%), Gulf Health Council (GHC) 91 (17%), Saudi Society for Clinical Nutrition (SSCN) 89 (16%), Kayl Association for Combating Obesity 83 (16%) and National Nutrition Committee (NNC) 80 (15%) and the lowest is Ministry of Health (MOH) 31 (5%). Significant relationships were observed between tweet accuracy and the source organization (*P*=.009, 95% CI 0.008‐0.01), content type (*P*=.03, 95% CI 0.03‐0.03), and tweet timing (*P*=.04, 95% CI 0.04‐0.04). Governmental sources had higher popularity and were more frequently accurate compared to nongovernmental sources.

**Conclusions:**

Reputable Twitter accounts in Saudi Arabia generally provide accurate nutrition-related content, though evidence citation is minimal. Users are encouraged to rely on reputable accounts for health information, and further research is suggested to explore the quality of evidence in such posts.

## Introduction

The global use of social media has increased exponentially, becoming one of the most popular online activities worldwide [1]. In 2020, over 3.6 billion people were using social media globally [2]. In line with this global trend, The Communications, Space, and Technology Commission (CST) has published the Saudi Internet Report 2022, indicating that there are 98.6% Internet use among the Saudi population [3]. The Ministry of Communications and Information Technology in Saudi Arabia reported a significant increase in the number of Saudi users of social programs and applications, rising from 8.5 million in 2015 to 18.3 million in 2020 [4]. Based on the Saudi Youth Statistic Report in 2023, there were 94.9 % of males and 94% of females participating on social networks [5]. Saudi Arabia was among the top 15 countries using Twitter (rebranded as X in July 2023), with over 12.45 million users [2,4], more than one-third of the Saudi population. Twitter ranks among the most popular social media platforms. It was launched in 2006 offering a free and interactive micro-blogging service where users create and share messages of up to 140 characters, called “tweets.” [6,7] There are over 500 million active users on Twitter alone, writing more than 340 million tweets and 1.6 billion search queries daily. Tweets can be posted via the web, instant online message, or mobile phone [6,7].

Social media is becoming a valuable tool for communication. Therefore, it is used in many fields, such as education, marketing, and health communication. The use of social media is limitless; some people use the platforms to receive medical consultations, while others use it to look for information related to their conditions [8]. Social media also provides a promising opportunity for public health promotion. For instance, social media platforms were used to deliver content as part of larger multiple component interventions for weight management [9]. It is considered a powerful tool for health education due to its ability to reach a large audience seamlessly, which makes it a practical and cost-effective tool to disseminate awareness content and messages [10,11]. Promoting awareness of dietary intake is vital for a healthy lifestyle and developing successful nutrition interventions. It is also considered the first step in population behavioral change [12,13]. Proper nutrition helps the body maintain healthy functions, growth, and reproduction and prevents diseases [12]. Participants mostly used Twitter to search for general knowledge, disease prevention and treatments for illness, nutrition, weight loss, and healthy lifestyle [8].

In recent years, patients have been engaging in unrestricted sharing of medical and health care information on social media [14]. Many patients use social media and online resources such as Facebook, Instagram, and Twitter to obtain health-related information to guide their health care decisions [14,15]. The widespread use and availability of social media in healthcare are attributed to several factors, such as the increase in ownership of mobile phones and the uptake of personal communication technologies worldwide [16]. The popularity of social media is perhaps due to its approachable and speedy nature and the ability to exchange user comments and feedback. In addition, governmental and health organizations have accounts on social media to disseminate news and health information to social media-using citizens.

According to the literature, the most popular platform studied for health-related information is Twitter [17]. Alassiri et al [8], mentioned that the number of Twitter users searching for health information in Saudi Arabia is increasing, two-thirds of the respondents preferred Twitter as a source due to its ease of use and accessibility. In Saudi Arabia, a study indicated that half of the participants considered Twitter their favorite social media platform, and a quarter of the participants indicated they preferred it to look for nutritional information [18].

Twitter is widespread and accepted in Saudi Arabia. The literature suggested that half of the Saudi Arabian population expressed a positive attitude toward seeking health information through Twitter [8]. Over a third of participants in a Saudi study expressed their satisfaction with using Twitter to find nutritional information [18]. Some participants reported having a better diet due to information available on Twitter [8] or that available health information on social media could enhance their health awareness [14].

Despite the numerous advantages that social media offers in the field of health communication, it has several challenging disadvantages. These challenges may include spreading falsified posts, misinformation, and the availability of massive amounts of information, which can create confusion among the general public [19,20]. Misinformed individuals can suffer from severe consequences that can lead to death in extreme cases as a result of social media rumors [20]. However, as the evidence suggests, the high number of tweets is not necessarily accurate [16,19]. A study indicated that tweets did not need to be accurate or evidence-based to gain traction [16]. While several studies have been conducted on the accuracy of health-related tweets, only 2 studies were conducted in Saudi Arabia by Albalawi et al [16] and Alnemer et al [6] focusing broadly on health, showing that at least half of the tweets are evidence-based. To our knowledge, no studies have specifically examined the content accuracy of nutrition-related tweets in Saudi Arabia, highlighting the gap that our study aims to address.

The study aims to evaluate the accuracy (evidence-based) of nutrition-related content on Twitter, posted by reputable Saudi Twitter accounts that are considered awareness providers disseminating nutritional awareness information in Saudi Arabia. Awareness providers are considered a reliable source of nutrition-related information, expecting their Twitter accounts and posts to hold the same level of reliability and accuracy. This study focuses on 7 Saudi Twitter accounts (governmental and nongovernmental) that provide and disseminate nutritional awareness content in the Kingdom of Saudi Arabia. Conducting such a study is a priority, considering the high popularity of Twitter in the Arab world.

## Methods

This study examined tweets and retweets in Arabic or English with diet or nutritional content posted on the official Twitter accounts of nutritional awareness providers in Saudi Arabia (governmental and nongovernmental). The study examined only official Twitter accounts affiliated with the entity to collect data.

This study performed a mixed methods content analysis of nutritional-related content published on Twitter. A semiqualitative approach was used to evaluate the content and accuracy of tweets. Twitter was chosen to investigate the nutrition-related content due to its popularity as a widely used social media platform in Saudi Arabia. This study followed a similar protocol to previous studies that assessed health-related content on Twitter [6,16]. A manual approach was used to identify and categorize nutritional-related tweets.

### Identification of Tweets

Manual data collection took place on the social media platform Twitter, including tweets in Arabic or English related to diet and nutrition from 7 selected nutrition awareness providers in Saudi Arabia. The process began with a preliminary search to identify official accounts disseminating nutritional information. This search reviewed publicly recognized accounts of health authorities, educational institutions, and professional organizations known for sharing nutrition-related content. The selection focused on highly relevant accounts, activity levels, and credibility. Tweets related to diet and nutrition that met the predefined inclusion criteria were included in the extraction sheet (see Multimedia Appendix 1 for the full extraction sheet).

### Criteria for Selecting Tweets (Inclusion Criteria)

The inclusion criteria for selecting tweets are as follows:

1. Any tweets focusing on nutrition-related content (text, media, graphics, or link to file).
2. Posted by 1 of the 7 nutrition awareness providers in Saudi Arabia that were identified by a preceding study. The study used 47 nutrition-related terms to search Twitter using the advanced search setting. The selection of the popular accounts was based on a minimum number of likes to 200, a minimum number of retweets to 200, and Saudi Arabia as a location. The following were identified as the most popular nutritional awareness providers in Saudi Arabia:

- Ministry of Health (MOH)
- Saudi Food and Drug Administration (SFDA)
- Gulf Health Council (GHC)
- Public Health Authority (Weqaya)
- Kayl Association for Combating Obesity
- Saudi Society for Clinical Nutrition (SSCN)
- National Nutrition Committee (NNC)
- Date of tweets starting from September 2020 until August 2021 (12-month period)

### Data Collection

A total of 8 data collectors manually identified and screened nutrition-related tweets published on the 7 official Twitter accounts within 12 months (September 2020 to August 2021). The extraction of tweets occurred in 2 phases.

#### Phase 1

Included the manual identification of tweets by 8 reviewers. In addition, 2 members independently reviewed each official account to identify the 400 most recent nutrition-related tweets and retweets. First, 1 member identified and collected all nutritional tweets on the account by adding them to the extraction sheet. Second, another reviewer went through the Twitter account and added any tweets not previously included in the extraction sheet (first screening). If there were less than 400 tweets, all nutrition-related tweets are included for the 12 months. The results of identified tweets by the 2 reviewers were combined to ensure the inclusion of all nutrition-related tweets. Finally, a third reviewer screened the extracted list of tweets to ensure that all included tweets met the inclusion and exclusion criteria (second screening). This phase produced a total of 814 selected nutrition-related tweets. See Table 1 for details on nutritional tweets data collection and screening.

**Table 1.** Nutrition-related tweets data collected and screened per account.

Phase	Names of entities							Total tweets (N=531)
	NNC^a^	SSCN^b^	Kayl^c^	Weqaya^d^	GHC^e^	SFDA^f^	MOH^g^	
Tweets collected, n	71	172	101	68	206	173	23	814
1st screening	+13	+18	+29	+10	+22	+21	+19	+132
2nd screening	−1	−10	−40	−13	−27	−31	−11	−133
Total of overall included tweets, n	83	180	90	65	201	163	31	813
Final randomly selected tweets^h^, n	83	100	90	65	100	100	31	569
Excluded tweets^i^, n	−3	−11	−7	−4	−9	−4	0	−38
Final selected tweets, n	80	89	83	61	91	96	31	

a NNC: National Nutrition Committee.

b SSCN: Saudi Society for Clinical Nutrition.

c Kayl: Kayl Association for Combating Obesity.

d Weqaya: Public Health Authority.

e GHC: Gulf Health Council.

f SFDA: Saudi Food and Drug Administration.

g MOH: Ministry of Health.

h The process used a simple random sampling technique to select 100 tweets from each of the 7 nutritional providers’ accounts in Saudi Arabia. The selection included all available tweets for accounts with fewer than 100 tweets.

i Duplicated, other languages, not found, or no information.

#### Phase 2

A simple random sampling technique was used to select 100 tweets from each of the 7 nutritional providers’ accounts in Saudi Arabia. The selection included all available tweets for accounts with fewer than 100 tweets. For the 3 accounts with more than 100 tweets (SSCN, GHC, and SFDA), unique identifiers were assigned to each tweet, and a random number generator selected 100 tweets from each account. This approach produced randomly selected 569 tweets using the same random number generator to ensure unbiased representation from the initial dataset of 813 tweets.

### Examination of Individual Tweets

The final 569 randomly selected diet and nutrition-related tweets were examined to collect data about the accounts and tweet characteristics. During the examination, we excluded 38 tweets due to duplication, languages, no longer available, or missing information.

A total of 531 tweets were examined and the following information was obtained: type of tweet (text, picture, video, voice, poll, and link to file), popularity (tweet likes, retweets, and number of comments), the topic of tweets (which was collected and then categorized into themes by analyzing the content of each tweet), and date and time of the tweet. From the account page on Twitter, the following information was collected: account activity or interaction with other Twitter users (following and followers), date of joining Twitter, and if there is a link to the organization. Each tweet was classified based on popularity (likes, retweets, and comments).

### Expert Opinion in Nutrition

A total of 3 independent experts from clinical nutrition, food science, and technology assessed the validity, accuracy, and evidence used in the nutrition-related tweets. Tweets were extracted on a Microsoft Excel file for all text tweets; links to files (primarily guides), and polls were converted to images. Original pictures, voice, and videos were cleaned to remove colors, logos, and identifiers. All tweets were provided as an Excel folder, including all tweets in a list, a separate folder labeled by the number of tweets for each tweet with media. These were provided to independent reviewers to give their expert opinion about all 531 tweets. Reviewers were blinded to the characteristics of tweets (original tweet or retweet) and the Twitter account during content analysis. In addition, a separate sheet was provided to rate tweets accurately. Rating was performed according to the following criteria:

1. Accuracy evaluation: The reviewers classified tweets as accurate or inaccurate. The score was decided based on most reviewers’ agreement [6]. If a tweet does not have a majority score agreement, then it will be considered inconclusive accuracy.
2. Evidence-based: If accurate, did the tweet include evidence or reference, link or reliable sources, or peer-reviewed studies or officially published guidelines?

### Sample Size

The sample size was estimated to find a significant difference in accuracy prevalence rate, and it was calculated based on a type one error of 0.05% and 80% power and a prevalence of outcome of 20 percent (according to Alnemer et al [6]) to be 304 tweets per awareness provider (account). Hence, it was aimed to collect a maximum of 400 most recent nutrition-related tweets by each of the 7 nutrition awareness providers in Saudi Arabia, published within 12 months for a total of 2800 tweets. A random selection of 100 tweets per account was made for the final sample to conduct the content analysis for accounts exceeding 100 tweets for validity and accuracy (the target total tweet number was 700 nutrition-related tweets). However, we could only include 531 tweets due to some accounts’ small number of nutritional tweets, such as of the MOH and Weqaya.

### Data Management

The collected data was analyzed using IBM SPSS (version 24.0). Nutrition-related tweets were categorized according to their accuracy level, account characteristics, and tweets. Descriptive statistics were used to tabulate frequencies and proportions, and the median of likes, comments, and retweets was computed. The chi-squared test (*χ*^*2*^) was used to determine statistical significance in assessing the difference between the accuracy of nutrition-related tweets; a *P* value<.05 is considered significant [6].

### Ethical Considerations

All Twitter user accounts and their identities were anonymized in this study. Only the affiliations of the 7 nutrition awareness providers in Saudi Arabia were identified. No personally identifiable data was included in the study. Data was stored securely in an encrypted, password-protected file. Ethical approval (IRBC/1481/21) was obtained from the Institutional Review Board office at King Abdullah International Medical Research Center (KAIMRC). This study does not require informed consent due to the public availability of the data collected from Twitter and its nature as publicly accessible information. While the data is publicly available, we ensured compliance with privacy and ethical standards by anonymizing all user information.

## Results

A total of 531 tweets posted by 7 different Saudi Arabian Twitter accounts were included in the study.

Table 2 illustrates some of the main characteristics of nutrition-related tweets. Of the 531 final tweets from 7 awareness providers, 279 (52.5%) were by governmental entities, and 252 (48%) were by nongovernmental entities. The majority of tweets, 96 (18%), were extracted from SFDA’s account, and the least number of tweets, 61 (12%) and 31 (5%) were extracted from Weqaya and MOH’s accounts, respectively. The rest of the entity’s accounts had almost the same number of tweets.

All accounts had an official website available on the Twitter page of the account except for SSCN, which accounts for 89 (17%) tweets published without an organizational website. Nearly half of the tweets, 219 (41.2%), were posted between March 2021 and May 2021, while the least number of tweets was between December 2020 and February 2021, 62 (12%).

Table 3 shows the number and proportion of tweets per month. April 2021 had the highest number of tweets, 113 (21.3%), while December 2020 and Feb 2021 had the lowest number of tweets, 18 (3%) in 12 months (see Table 3). Of the 531 tweets, 262 (49.3%) were published from 4 PM to 11:59 PM, and 233 (43.9%) were published from 8 AM to 3:59 PM. In contrast, only 36 (7%) tweets were published between 12 AM and 7:59 AM.

**Table 2.** Descriptive statistics of the nutritional tweets (N=531).

Variables	Tweets (N=531), n (%)
Account	
SFDA^a^	96 (18)
GHC^b^	91 (17)
SSCN^c^	89 (17)
Kayl^d^	83 (16)
NNC^e^	80 (15)
Weqaya^f^	61 (12)
MOH^g^	31 (5)
Type of organization	
Governmental	279 (52.5)
Nongovernmental	252 (47.5)
Organization link	
Yes	442 (83.2)
No	89 (17)
Date	
September 2020 to November 2020	142 (26.7)
December 2020 to February 2021	62 (12)
March 2021 to May 2021	219 (41.2)
June 2021 to August 2021	108 (20.3)
Time	
12 AM to 7:59 AM	36 (7)
8 AM to 3:59 PM	233 (43.9)
4 PM to 11:59 PM	262 (49.3)
Topic	
Diet or eating habits	173 (32.6)
Nutrition label, nutrients, or supplements	122 (23)
Disease or condition	109 (20.5)
Food safety	49 (9)
Ramadan	60 (11)
Water or dehydration	18 (3)
Type of content	
Text	90 (17)
Picture	342 (64.4)
Video	67 (13)
Voice	11 (2)
Poll	7 (1)
Link to file	14 (3)
Accuracy	
Accurate	445 (83.8)
Inaccurate	13 (2)
Inconclusive accuracy	73 (14)
Evidence-based	
With evidence	17 (4)
Without evidence	428 (96.2)

a SFDA: Saudi Food and Drug Administration.

b GHC: Gulf Health Council.

c SSCN: Saudi Society for Clinical Nutrition.

d Kayl: Kayl Association for Combating Obesity.

e NNC: National Nutrition Committee.

f Weqaya: Public Health Authority.

g MOH: Ministry of Health.

**Table 3.** Number and proportion of tweets by month for the 12-month period (N=531).

Month-year tweeted	n Tweets (N=531), n (%)	
September 2020	64 (12)	
October 2020	39 (7)	
November 2020	39 (7)	
December 2020	18 (3)	
January 2021	26 (5)	
February 2021	18 (3)	
March 2021	52 (10)	
April 2021	113 (21.3)	
May 2021	54 (10)	
June 2021	41 (8)	
July 2021	42 (8)	
August 2021	25 (5)	

Most of the tweets’ topics were about diet or eating habits, accounting for 173 (32.6%); nutrition labels, nutrients, or supplements 122 (23%), and diseases/conditions 109 (20.5%). In contrast, the least tweeted topic was about food safety at 49 (9%), Ramadan at 60 (11%), and water or dehydration at 18 (3%). Most tweets were in picture content 342 (64.4%), followed by texts 90 (17%). On the other hand, voices, polls, and links were all less than 10%. More than 3-quarters of the tweets, 445 (83.8%), were accurate, whereas 13 (2%) tweets were inaccurate, and 73 (14%) had inconclusive accuracy (no agreement) based on the expert’s opinion. Among accurate tweets, 17 (4%) were evidence-based (included a mention of the evidence in the tweet), and 428 (96.2%) were not evidence-based.

The popularity of tweets based on likes, comments, retweets, and followers for each account is shown in Table 4. The median of tweet likes was 14 likes per account (range 0‐1442). The median of retweets was 8 (range 0-352) per account, and the median of comments was 1 (range 0‐133) per account. Finally, the median number of followers per account was 158,600 (range 1656-6,000,000).

Table 5 shows crosstabulation results describing the correlation between the accuracy of tweets and all characteristics using *Χ*^*2*^ and fisher exact tests. There were significant associations between accuracy of tweets and the following characteristics: inclusion of evidence (*P*<.01, 95% CI <0.001-<0.001), source (account; *P*=.009, 95% CI 0.008‐0.01), type of organization (*P*=.009, 95% CI 0.007‐0.01), time tweeted (*P*=.04, 95% CI 0.04‐0.04), topic of tweets (*P*=.003, 95% CI 0.002‐0.004), and type of content (*P*=.03, 95% CI 0.03‐0.03).

A total of 428 (80.6%) accurate tweets did not include or mention the source of evidence. Another significant relation was found between the accuracy of the tweets and the source of data (accounts). The SFDA account had the highest number of accurate tweets, 92 (17%), while MOH had the lowest number of accurate tweets, 28 (5%). The GHC had the highest number of not-accurate tweets with 5 (1%), whereas MOH had the lowest with 0 inaccurate tweets. A relationship was also found between the accuracy of tweets and the type of organization. Nongovernmental organizations had more tweets with inconclusive accuracy (n=46, 9%). There is also a relationship between accuracy and the time tweeted and between the accuracy of the tweets and the topic tweeted. Diet and eating habits had the highest number of accurate tweets, 143 (26.9%), followed by disease and conditions, 95 (18%). Yet, nutrition labels, nutrients, or supplements had the highest number of inaccurate tweets at 8 (2%). Furthermore, there was a significant association between the accuracy of the tweets and the type of published content. The most accurate tweets were pictured, 291 (54.8%). No significant differences were found between the accuracy of the tweets and the rest of the variables (organizations, link, date, number of likes, comments, and retweets).

Table 6 shows the popularity by account based on a score of the average likes, comments, and retweets and the number of account followers. The MOH had the highest rank, making it the most popular account, followed by SFDA and GHC. The NNC and SSCN had the least popularity among all accounts.

**Table 4.** Median likes, comments, retweets, and followers.

Metric	Values, median (IQR)
Likes	14 (0‐1422)
Comments	1 (0‐133)
Retweets	8 (0‐352)
Followers	158,600 (1656-6,000,000)

**Table 5.** Cross-tabulation showing the correlation between the accuracy of the nutritional tweets and characteristics (N=531).

Variables	Accurate, n (%)	Inaccurate, n (%)	Inconclusive accuracy, n (%)	*P* value	95% CI
Included evidence^a^				<.001	<.001 to <.001
Yes	17 (3)	0 (0)	0 (0)
No	428 (80.6)	0 (0)	0 (0)
Not applicable	0 (0)	13 (2)	73 (14)
Account^a^				.009	.008-.01
GHC^b^	72 (14)	5 (0.9)	14 (3)
Kayl^c^	65 (12)	1 (0.2)	17 (3)
MOH^d^	28 (5)	0 (0.0)	3 (0.6)
NNC^e^	65 (12)	2 (0.4)	13 (2)
SFDA^f^	92 (17)	1 (0.2)	3 (0.6)
SSCN^g^	72 (14)	1 (0.2)	16 (3)
Weqaya^h^	51 (10)	3 (0.6)	7 (1)
Type of organization				.009	.007-.01
Governmental	243 (45.8)	9 (1.7)	27 (5)
Nongovernmental	292 (38)	4 (0.8)	46 (9)
Organization link				.34	.33-.35
Yes	373 (70.2)	12 (2.3)	57 (11)
No	72 (14)	1 (0.2)	16 (3)
Date^a^				.96	.95-.96
September 2020 to November 2020	117 (22)	3 (0.6)	22 (4)
December 2020 to February 2021	52 (10)	1 (0.2)	9 (2)
March 2021 to May 2021	185 (35)	5 (0.9)	29 (6)
June 2021 to August 2021	91 (17)	4 (0.8)	13 (2)
Time^a^				.04	.04-.04
12 AM to 7:59 AM	25 (4.7)	3 (0.6)	8 (2)
8 AM to 3:59 PM	202 (38)	3 (0.6)	28 (5)
4 PM to 11:59 PM	218 (41.1)	7 (1.3)	37 (7)
Topic^a^				.003	.002-.004
Diet or eating Habits	143 (26.9)	4 (0.8)	26 (5)
Nutrition labels, nutrients, or supplements	92 (17.3)	8 (1.5)	22 (4)
Disease or condition	95 (17.9)	1 (0.2)	13 (2)
Food safety	49 (9.2)	0 (0)	0 (0)
Ramadan	52 (9.8)	0 (0)	8 (2)
Water or dehydration	14 (2.6)	0 (0)	4 (1)
Type of content^a^				.03	.03-.03
Text	64 (12.1)	3 (0.6)	23 (4)
Picture	291 (54.8)	9 (1.7)	42 (8)
Video	60 (11.3)	0 (0)	7 (1)
Voice	11 (2.1)	0 (0)	0 (0)
Poll	6 (1.1)	0 (0)	1 (0.2)
Link to file	13 (2.4)	1 (0.2)	0 (0)

a Fisher exact test.

b GHC: Gulf Health Council.

c Kayl: Kayl Association for Combating Obesity.

d MOH: Ministry of Health.

e NNC: National Nutrition Committee.

f SFDA: Saudi Food and Drug Administration.

g SSCN: Saudi Society for Clinical Nutrition.

h Weqaya: Public Health Authority.

**Table 6.** Popularity ranking by account score based on the average number of retweets, likes, comments, and the number of account followers (rank of 1 indicates the highest and 6 indicates the lowest popularity).

Account	Average comment		Average retweet		Average like		Follower per account		Total score of rank	Rank of popularity
	Rank	n	Rank	n	Rank	n	Rank	n		
MOH^a^	1	17.26	1	138.58	1	230.19	1	6,000,000	4	1
SFDA^b^	2	6.11	2	45.25	3	61.24	2	1,300,000	9	2
GHC^c^	3	4.88	3	32.95	2	103.60	3	294,800	11	3
Weqaya^d^	7	0.91	4	11.53	4	18.63	4	158,600	19	4
Kayl^e^	6	1.61	5	10.05	5	15.54	5	12,400	21	5
NNC^f^	5	1.76	6	7.93	7	11.39	6	5657	24	6
SSCN^g^	4	2.02	7	7.09	6	14.93	7	1656	24	6

a MOH: Ministry of Health.

b SFDA: Saudi Food and Drug Administration.

c GHC: Gulf Health Council.

d Weqaya: Public Health Authority.

e Kayl: Kayl Association for Combating Obesity.

f NNC: National Nutrition Committee.

g SSCN: Saudi Society for Clinical Nutrition.

## Discussion

### Principal Findings

This study aimed to assess the validity of nutrition-related content posted on Twitter by 7 reputable Saudi Arabian Twitter accounts that disseminate nutritional awareness information in Saudi Arabia. Both governmental and nongovernmental organizations were included in our analysis. The validity of nutrition-related tweets posted on Twitter by preidentified Saudi Arabian nutritional awareness providers was examined, and deficiencies and gaps in nutrition-related content on Twitter were identified.

This study’s findings indicated that most tweets (84%) were deemed accurate based on experts’ opinions. Albalawi et al [16] examined the trustworthiness of tweets and found that roughly 69% of health-related tweets were trustworthy. Yet, Alnemer et al [6] found that approximately half of the extracted health-related tweets (49%) were evidence-based. Furthermore, the findings of Lee et al [21] indicate that over half of health-related tweets (53%) contained “testable” claims supported by medical evidence. Public’s tweets were less accurate than health care professional’s tweets [22]. These results, both locally and internationally, are somewhat consistent with the findings of this study. Yet, the number of accurate tweets is significantly higher in this study, 84%, compared to Alnemer et al [6], Albalawi et al [16], or Lee et al [16]. This might be due to the preselection of reputable awareness providers. Therefore, the information tweeted was of higher quality. It is worth noting that all previous studies focused on health-related tweets, yet we focused on nutrition-related tweets in this study. In addition, this study included 3 levels of accuracy (accurate, inaccurate, and inconclusive accuracy), whereas in the previous studies, they only classified tweets as accurate or inaccurate.

Among accurate tweets, only 4% of tweets cited or mentioned the source of the evidence. This might be due to the limited capacity of allowed characters per tweet. Likewise, previous studies conducted locally found that a significant number of the extracted tweets were classified as untrustworthy health information [6,16]. However, previous studies only classified tweets based on accuracy (the same as evidence-based); in this study, we also looked at including the source of evidence in the tweet to allow readers to verify or read more about the topic. Therefore, including evidence in our study has a different meaning than evidence based on previous studies [6,16,21]. Yet, this highlights that low inclusion of references in tweets is a common challenge, which might be due to the limited characters allowed on the platform. To overcome this challenge, content creators could use hyperlinks, multimedia (pictures and videos) [23], or threading tweets [24] to accommodate references without exceeding character limits.

In addition, Alassiri et al [8] reported that over half of their study population doubted the credibility of information available on Twitter, which contradicts this study’s findings regarding the reliability of shared information. According to our experts, only 2% of tweets were deemed inaccurate. Again, this discrepancy might be explained by the preselection of reputable awareness providers to include in this study rather than selecting any nutritional-related tweets. Therefore, the information tweeted might be of higher quality by professionals in the field.

The study’s findings also revealed that 13% of the tweets did not have a majority agreement on content accuracy by experts. Although this number is relatively small, it is an interesting finding. A possible explanation might be the influence of reviewers’ backgrounds and affiliations with different schools of nutrition. Our reviewers had backgrounds in clinical nutrition, food science, and food science technology, which may have affected their evaluation of the tweets. Their differing expertise may have influenced their evaluations, highlighting the challenge of ensuring reliable nutrition content on social media. This variation in expert opinions emphasizes the need for consistent, evidence-based guidelines to improve the quality of online nutrition information. To improve consistency and reliability of the evaluation, standardized international or national nutritional guidelines could be used as a reference for evaluating content [25,26]. In addition, using consensus decision techniques such as the Delphi method could produce agreement among a group of experts in future studies [27]. This finding matches other studies in the literature; Albalawi et al [16] and Alnemer et al [6] identified conflicting opinions between study reviewers regardless of the evaluation. These findings suggest that in nutrition, conflicting opinions arise within the field of diet and nutrition, which can impact the interpretation and analysis of the tweets. This also highlights the importance of considering different perspectives and sources of information when analyzing social media content related to diet and nutrition. Therefore, it is essential to recognize the potential influence of individual biases and perspectives when analyzing social media content.

The study results indicate a significant relationship between the accuracy of tweets and the source of data (accounts). Based on expert opinions, the majority of tweets were accurate in governmental and nongovernmental organizations, with 2% inaccurate. As expected, the MOH was the most popular account, yet had the lowest number of nutritional tweets with zero inaccurate tweets. Meanwhile, the SFDA, which also represents a government organization, had the highest number of accurate tweets, followed by the GHC. Yet, nongovernmental organizations had a higher number of tweets with inconclusive accuracy. These findings were consistent with those reported by Alnemer et al [6], who found that 80% of government institute tweets were valid, and by Albalawi et al [16], who found that organizational accounts and traditionally trusted health sources were more likely to tweet accurate information. Also, the study by Lee et al [21] indicated that health care organizations such as hospitals, medical societies, health care clinics, and journals were more likely to share “testable” claims, which were expected to be supported by medical evidence, than other accounts. However, in this study, we only included reputable awareness providers who are known in health and nutrition.

The SSCN and Kayl, representing associations considered nongovernmental organizations mainly focused on nutrition, had the third and fourth highest number of accurate tweets despite being among the least popular accounts, ranked 6 and 5, respectively (see Table 5). These findings suggest that government and nongovernment organizations are reliable and trustworthy, regardless of their popularity. Although the number is small, it is notable that governmental organizations represented by the GHC had the highest number of inaccurate tweets (5, 1%), followed by Weqaya and NNC, which are governmental organizations. It is worth noting that although Weqaya had the second-lowest number of tweets related to nutrition, it had the highest number of inaccurate tweets. This highlights the possibility that even trusted health sources may publish inaccurate nutrition-related information, especially if they are nonspecialized entities in nutrition. This highlights the importance of critically evaluating nutrition information from social media platforms. While it is encouraging that government and nongovernment organizations were found to be reliable and trustworthy overall, inaccurate information underscores the need for continued efforts to ensure the accuracy and reliability of nutrition-related content on social media.

In addition, the MOH had the lowest number of nutritional tweets, which may be attributed to the relatively low number of nutrition-specific tweets posted by the account. MOH is considered one of the main awareness providers; it frequently posts about a variety of topics related to health. Its Twitter account covers a wide range of topics, including public health campaigns, disease prevention, and general health care updates especially during the pandemic. As a result, nutrition-related content may not receive as much focus or prioritization as other health topics such as COVID-19 topics. Given the timing during the COVID-19 pandemic, the MOH account focused more on evidence-based information in response to the pandemic and increased demand for health-related content [28]. The public’s heightened concern for reliable health information during the global health crisis could have influenced the nature of the tweets [28]. Also, MOH may have fewer resources or a smaller team dedicated explicitly to creating nutrition-related content compared to specialized organizations such as SFDA or NNC. Although there is limited focus on nutrition, it is suggested that MOH has a good tweet quality shared through their account. In addition, the dehydration topic had the lowest tweets, followed by food safety and Ramadan. While Ramadan-related topics are usually tweeted in the holy month, the same is not expected for dehydration and food safety. Therefore, nutritional topic priorities should be identified on a national level to be shared with awareness providers to guide creating their content and avoid underrepresentation of specific topics.

A significant association was observed between the accuracy of tweets and their topic, classified into 6 categories: diet or eating habits; nutrition label, nutrients, or supplements; disease or condition; food safety; Ramadan, and water or dehydration. Alassiri et al [8] reported that most of their study population held a favorable attitude toward using Twitter, particularly concerning nutrition and healthy lifestyles. It is encouraging to note that nutrition awareness providers in Saudi Arabia are publishing a high number of tweets related to diet or eating habits and nutrition labels, nutrients, or supplements, which are topics of particular interest to Twitter users, based on the study by Alassiri and Alowfi [8]. However, it is equally essential to ensure that information across all categories is accurate and trustworthy, as inaccurate data can seriously affect individuals and society [8].

Another significant finding was the observed association between tweet accuracy and the type of content shared. Tweets presented in picture format had a higher likelihood of being accurate compared to text-only tweets. This association might be because pictures allow for more comprehensive and visually organized information, which reduces the risk of oversimplification or misrepresentation often observed in text-constrained formats [29,30]. In addition, pictures provide an opportunity to include detailed content, such as diagrams or infographics, which can enhance accuracy by presenting evidence in a visually structured way. Twitter’s character limit likely drives the preference for visual content as it enables more efficient communication [29,30]. Beyond accuracy, visual content is generally more engaging and attractive than other formats. Pictures effectively transmit information and help users understand and remember the content [29]. Furthermore, their ability to spread quickly and perform better on social media platforms enhances their reach and impact [29,30]. Pictures can also be more appealing specifically to individuals with lower health literacy [23].

No significant difference was found between the accuracy of tweets and the time they were posted. However, it is noteworthy that most of the accurate tweets with evidence were posted between 8 AM and 3:59 PM, despite the majority of tweets being posted at night. This finding is in some way matching with those of Albalawi et al [16], who found that the majority of trustworthy tweets were posted during the daytime.

No significant differences were found between the accuracy of extracted tweets and their average number of retweets and favorites. This finding is consistent with the results reported by Albalawi et al [16], although accurate tweets were more likely to be liked and retweeted by others in their study. A study conducted by Hand et al [31] discovered that the source of tweets has a significant impact on its dissemination and popularity. The study found that tweets extracted from professional, government, patient advocacy organizations, or charities were more likely to be marked as favorites and retweeted compared to tweets from other sources [31]. This finding is (in some way) in agreement with this study, which has also shown that governmental accounts tend to be more popular than nongovernmental (nonprofit) accounts. It is important to note that the study by Hand et al [31] was conducted within a specific context and within a particular location and population, which may introduce bias into the comparison.

It is important to bear in mind that the studies conducted by Albalawi et al [16], Alnemer et al [6], and Lee et al [21], all focused on the analysis of health-related tweets, while Hand et al [31] analyzed social media content related specifically to nutrition and heart failure. In contrast, this study exclusively examined nutrition-related tweets. This distinction is important to be noted when comparing the results of these studies, as the research question and the topic of analysis can significantly impact the findings.

Marar et al [14] reported that a significant number of study participants not only sought health information through social media but also found it reliable and believed it increased their health awareness and knowledge. Furthermore, over 3-quarters of participants stated that this information had an impact on their health status [14]. The findings reported here suggest that there are varying perspectives on the accuracy and reliability of nutrition or health-related content on Twitter. These highlight the importance of critically evaluating health information obtained from social media and seeking out multiple sources to ensure the information is evidence-based and reliable. Furthermore, as Marar et al [14] reported, the influence of social media on health-related knowledge and behavior is substantial, indicating the need for continued efforts to ensure the accuracy and reliability of health information disseminated through these channels. In general, these results assure the importance of promoting evidence-based nutrition information on social media and the need for consumers to approach health-related content with a critical eye.

### Conclusions

In conclusion, based on our analysis, nutrition-related tweets posted by reputable awareness providers are considered mostly accurate. Yet, inclusion of the source of evidence in the tweet was not a common practice, although it may enhance the user’s experience and knowledge. To enhance the reliability of nutrition content on social media, we recommend implementing citation guidelines for health-related accounts, such as providing links to credible sources and adopting recognized citation formats by using hyperlinks and multimedia. These practices would increase transparency and trustworthiness, benefiting both content creators and users.

The results indicate that regardless of the type of organization, time, date, topic, or type of content tweeted, if the source tweeting is knowledgeable and reputable, then tweets are more likely to be accurate. Also, nutrition-related tweets have a different content type, while there is a clear preference to tweeting pictures and text. It is also observed that there are diverse topics, yet there is a clear need to balance the distribution of topics. For instance, more tweets about food safety and dehydration. These results are reassuring that social media can have accurate and evidence-based information. However, users should be selective in choosing the Twitter accounts to seek for information.

This study offers opportunities for further research, including subanalysis of accuracy levels and quality of evidence. Also, future studies could use automation tools and software such as social media APIs and machine learning to automate the identification, extraction, and analysis of tweets to improve data collection efficiency. These tools could also be used to link content creators and cross-referencing their qualifications with tweets. This will overcome the manual analysis challenges such as resource-intensive and human error.

### Limitations

This study has few limitations, while the data collection started on September 2021, some of the tweets were a year old (such as posted in September 2020). This means that the landscape of tweets might have changed over time. The data collection was performed manually; although the data were screened multiple times to control for any fallibility, there is still a chance of errors and mistakes. Also, along the process of extracting tweets, some tweets were no longer available, which might have different characteristics than the included tweets. For some users (Kayl, NNC, Weqaya, and MOH), there were not enough number of tweets to randomly select; therefore, all tweets were included for these accounts. Therefore, accounts did not have an equal number of nutritional tweets to compare between.

## Supplementary material

10.2196/68128 Multimedia Appendix 1 Extraction sheet for tweets.

## Abbreviations

GHC: Gulf Health Council
MOH: Ministry of Health
NNC: National Nutrition Committee
SFDA: Saudi Food and Drug Administration
SSCN: Saudi Society for Clinical Nutrition
