# Correction: Accuracy of Nutrition-Related Awareness Messages on Twitter (Rebranded as X) by the Nutrition Awareness Providers in the Kingdom of Saudi Arabia: Validity Content Analysis

**DOI:** 10.2196/85577

**Published:** 2025-10-23

**Authors:** Duaa Alammari, Iman A Bindayel, Noura Althukair, Noura AlRomi, Khalid Aldubayan, Najla Khateeb, Ahmed Alabdrabalnabi, Banan Banamah

**Affiliations:** 1 Department of Health Systems Management, College of Public Health and Health Informatics, King Saud Bin Abdulaziz University for Health Sciences, Prince Mutib Ibn Abdullah ibn Abdulaziz Rd, Riyadh, 13213, Saudi Arabia, 966 500844881; 2 King Abdullah International Medical Research Center, Riyadh, Saudi Arabia; 3 Department of Community Health and Sciences, College of Applied Medical Sciences, King Saud University, Riyadh, Saudi Arabia; 4 College of Applied Medical Sciences, King Saud Bin Abdulaziz University for Health Sciences, Al Ahsa, Saudi Arabia; 5 Department of Public Health, College of Health Sciences, Saudi Electronic University, Dammam, Saudi Arabia

In “Accuracy of Nutrition-Related Awareness Messages on Twitter (Rebranded as X) by the Nutrition Awareness Providers in the Kingdom of Saudi Arabia: Validity Content Analysis” [1], the authors noted one error.

Author AA’s affiliation was originally published as:

Department of Clinical Nutrition, College of Health Sciences, Saudi Electronic University, Dammam, Saudi Arabia

This has now been corrected to:

Department of Public Health, College of Health Sciences, Saudi Electronic University, Dammam, Saudi Arabia

The correction will appear in the online version of the paper on the JMIR Publications website, together with the publication of this correction notice. Because this was made after submission to PubMed, PubMed Central, and other full-text repositories, the corrected article has also been resubmitted to those repositories.
