# Supplementary material for: Accuracy of Nutrition-Related Awareness Messages on Twitter (Rebranded as X) by the Nutrition Awareness Providers in the Kingdom of Saudi Arabia: Validity Content Analysis
Source: Online J Public Health Inform. 2025 Sep 26;17:e68128. doi: 10.2196/68128 (PMC12468160; doi:10.2196/68128)
Supplement: Multimedia Appendix 1 [file ojphi-v17-e68128-s001.docx]

**Appendix A. Extraction sheet for tweets**
